# Greensporone C, a Freshwater Fungal Secondary Metabolite Induces Mitochondrial-Mediated Apoptotic Cell Death in Leukemic Cell Lines

**DOI:** 10.3389/fphar.2018.00720

**Published:** 2018-07-16

**Authors:** Kirti S. Prabhu, Kodappully Sivaraman Siveen, Shilpa Kuttikrishnan, Ahmad N. Iskandarani, Abdul Q. Khan, Maysaloun Merhi, Halima E. Omri, Said Dermime, Tamam El-Elimat, Nicholas H. Oberlies, Feras Q. Alali, Shahab Uddin

**Affiliations:** ^1^Translational Research Institute, Academic Health System, Hamad Medical Corporation, Doha, Qatar; ^2^National Center for Cancer Care and Research, Hamad Medical Corporation, Doha, Qatar; ^3^Department of Medicinal Chemistry and Pharmacognosy, Faculty of Pharmacy, Jordan University of Science and Technology, Irbid, Jordan; ^4^Department of Chemistry and Biochemistry, University of North Carolina at Greensboro, Greensboro, NC, United States; ^5^College of Pharmacy, Qatar University, Doha, Qatar

**Keywords:** AKT, apoptosis, greensporone C, leukemia, reactive oxygen species

## Abstract

Therapeutic agents used in the treatment of cancer are known to develop resistance against cancer cells. Hence, there is a continuing need to investigate novel agents for the treatment and management of cancer. Antitumor activity of greensporone C (GC), a new resorcylic acid lactone isolated from an organic extract of a culture of a *Halenospora* sp. freshwater fungus, was subjected for screening against a panel of leukemic cell lines (K562, U937, and AR320). In all the three cell lines, cell proliferation was inhibited in dose-dependent fashion. GC further arrested the cells in SubG0 phase in dose-dependent manner. Annexin V/PI dual staining data confirmed apoptotic death of treated K562 and U937 leukemic cells. Treatment with GC suppressed constitutively phosphorylated AKT and downregulated expression of inhibitor of apoptotic proteins XIAP, cIAP-1, and cIAP-2. In summation to this, GC-treated leukemic cells upregulated protein expression of pro-apoptotic proteins, Bax with concomitant decrease in expression of anti-apoptotic proteins including Bcl-2 and Bcl-xL. Upregulation of Bax was associated with cytochrome c release which was confirmed from the collapse of mitochondrial membrane. Released cytochrome c further activated caspase cascade which in turn initiated apoptosis process. Anticancer activity of this isolated fungal compound GC was potentiated via stimulating production of reactive oxygen species (ROS) along with depletion of reduced glutathione (GSH) levels in K562 and U937 leukemic cells. Pretreatment of these cells with *N*-acetyl cysteine prevented GC-induced depletion of reduced GSH level and mitochondrial-caspase-induced apoptosis. Altogether, our data show that GC modulates the apoptotic response of human leukemic cells and raises the possibility of its use as a novel therapeutic strategy for hematological malignancies.

## Introduction

Natural products are and have been playing vital role in the area of drug discovery. In the last ∼75 years, out of 175 new entities that were labeled as anti-cancer 49% were isolated from natural products (Newman and Cragg, 2016). Plantae, eubacteria, and fungi are the major kingdoms of life to provide secondary metabolites. From approximately 0.5 million secondary metabolites (natural products) that have been described to date, about 14% (70,000) were of microbial origin. Moreover, of about 33,500 bioactive microbial natural compounds, 47% were of fungal origin (Bills and Gloer, 2016). Fungal secondary metabolites have contributed immensely to the drug discovery process by providing many novel drugs, including β-lactam antibiotics (penicillin G), cholesterol-lowering agents (lovastatin), and immunosuppressant (fingolimod) (Fleming, 1946; Vagelos, 1991; Strader et al., 2011). Recently, fungal compounds have been gaining a lot of importance and recognition in the area of anti-cancer drug discovery world-wide with some being currently under investigation in different phases of clinical trials (Kinghorn et al., 2016). From estimated 5.1 million species of fungi on earth (Blackwell, 2011), only about 99,000 species have been described (Blackwell, 2011), and a smaller fraction of these were explored for bioactive secondary metabolites (Kinghorn et al., 2016). Freshwater fungi, which flourishes in freshwater ecosystems and is primarily involved in the decomposition of submerged plant debris represents an even less studied area in mycology with only about 3,000 species being identified so far (Jones et al., 2014).

As part of our research to explore the chemical diversity of freshwater fungi, a series of 14 resorcylic acid lactones were isolated and identified from an organic fraction of a freshwater fungal isolate *Halenospora* sp. Among the isolated compounds, greensporone C (GC) showed promising cytotoxic activity when examined against the MDA-MB-435 (breast cancer) and HT-29 (colon) cancer cell lines, with IC_50_ values of 2.9 and 7.5 μM, respectively (El-Elimat et al., 2014). Macrocyclic compounds due to their vivid pharmacological activities and better bioavailability have gained growing interest in the area of drug discovery (Giordanetto and Kihlberg, 2014).

Our study aimed to understand the mechanism of GC-mediated cytotoxic effects using a series of leukemic cells as model. GC-treated K562 and U937 cells underwent apoptosis which was mediated by inhibition of uncontrolled cell growth by downregulating protein expression of constitutively activated AKT. Inhibitor of apoptosis proteins (IAPs) known to be downstream targets of AKT along with various antiapoptotic proteins such as Bcl-2, Bcl-xL, etc. were also downregulated favoring mitochondrial-caspase-mediated apoptosis. In addition, GC-mediated cytotoxic effects are mediated by generation of ROS. Our findings strongly suggest that GC has a strong potential to become a promising lead compound in the treatment of leukemic cells and in other human cancers where PI3-kinase/AKT pathways are constitutively activated.

## Materials and Methods

### Isolation of Greensporone C (GC) From Aquatic Fungi

**FIGURE 1:**
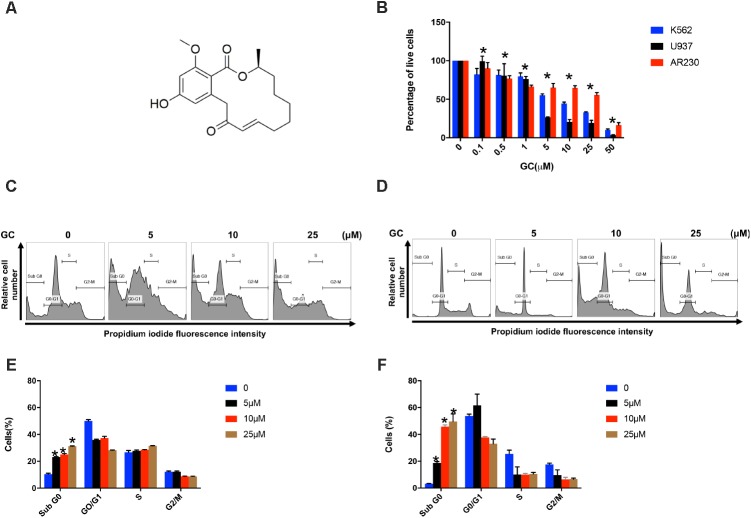
Effects of GC on cell proliferation and cell cycle. **(A)** Chemical structure of GC. **(B)** Inhibition of cell viability was measured using MTT assay as described under the section “Materials and Methods.” Cell cycle fraction analysis of cells in response to GC. K562 **(C)** and U937 **(D)** cells were treated with GC as indicated and analyzed by flow cytometry. GC significantly enhanced SubG0 fraction in K562 **(E)** and U937 **(F)**. The graph displays the mean ±SD of three independent of experiments. ^∗^*P* < 0.05.

Greensporone C was isolated from a chloroform:methanol (1:1) extract of a culture of an aquatic fungus (G87) that was samples from a submerged woody substrate collected from a stream on the campus of the University of North Carolina at Greensboro. The organic extract was further subjected to liquid–liquid partitioning and then to a series of fractionation and purifications procedures, including normal-phase flash chromatography and reversed-phase preparative and semi-preparative HPLC. The structure of GS was identified using various spectroscopic and spectrometric techniques, including HRESIMS, 1D-NMR (^1^H and ^13^C), and 2D-NMR (COSY, edited-HSQC, and HMBC). The absolute configuration of the stereogenic center (C-2) was established as 2*S*. The purity of GC was measured using UPLC and found to be >98% (**Figure 1A**; El-Elimat et al., 2014).

### Chemicals and Reagents

Antibodies viz., caspase-9, caspase-8, caspase-3, cleaved-caspase-3, poly(ADP-ribose) polymerase (PARP), XIAP, cIAP-1, cIAP-2, Bcl-2, Bcl-xL, and Bax were procured from Cell Signaling Technologies (Beverly, MA, United States) and GAPDH antibody was purchased from Santa Cruz Biotechnology, Inc. (Santa Cruz, CA, United States). Annexin V-FITC, propidium iodide staining solution, Hoechst 33342 Solution, BD Cytofix/Cytoperm plus fixation and permeabilization solution kit, and BD MitoScreen (JC-1) Kit were purchased from BD Biosciences (NJ, United States). CCK-8 kit and *N*-acetyl cysteine (NAC) was obtained from Sigma-Aldrich (St. Louis, MO, United States). z-VAD-FMK was purchased from Calbiochem (San Diego, CA, United States). CellROX Green, MitoSOX Red, and ThiolTracker Violet were purchased from Invitrogen (MA, United States).

### Cell Culture

The leukemic cells K562, U937, and AR230 were grown as described previously (Iskandarani et al., 2016) in RPMI 1640 medium supplemented with 10% fetal bovine serum (FBS), 100 U/ml penicillin, and 100 U/ml streptomycin at 37°C in a humidified atmosphere containing 5% CO_2_. Peripheral blood mononuclear cell (PBMC) was obtained from healthy donors after informed consent and with ethical approval (Study number: 16062/16) from the Institutional Review Board, Hamad Medical Corporation, Doha, Qatar. PBMCs were separated with Ficoll-Paque-based density centrifugation and then cultured in RPMI 1640 medium supplemented with 10% FBS, 100 U/ml penicillin, and 100 U/ml streptomycin at 37°C in a humidified atmosphere containing 5% CO_2_.

### Cell Proliferation Assay

The panel of leukemic cell lines treated in presence and absence GC was subjected for evaluating cell viability by using CCK-8 colorimetric method. The amount of formazan dye formed by reduction of WST-8 salt [2-(2-methoxy-4-nitrophenyl)-3-(4-nitrophenyl)-5-(2,4-disulfophenyl)-2H-tetrazolium] by dehydrogenase present in cells is directly related to the presence of viable cells. In brief, 1 × 10^4^ cells per well were plated in 96-well microtiter plates and treated with indicated doses of GC for 24 h. At the end of 24 h, as per manufacturer protocol CCK-8 solution was added and plates were read at 450 nm. Percentage of cell viability was calculated as OD of the experiment samples/OD of the control sample × 100 (Wang et al., 2018).

### Cell Cycle Analysis

K562 and U937 were treated with escalating concentrations of GC for 24 h. Cell cycle analysis was performed using Hoechst 33342 and cell cycle fractions were analyzed by flow cytometry BD LSRFortessa analyzer (BD Biosciences, NJ, United States) (Siveen et al., 2014).

### Annexin V/Propidium Iodide Dual Staining

K562 and U937 cells were treated with various doses of GC as indicated for 24 h. Cells were washed with PBS and stained with fluorescein-conjugated annexin-V and propidium iodide in 1× annexin binding buffer for 20 min. Flow cytometry was used to quantify cells that were either viable or had undergone apoptosis or necrosis after treatment (Badmus et al., 2015). Percentage apoptosis was expressed as a combination of cells present in early and late apoptosis (Prabhu et al., 2017).

### Cell Lysis and Immunoblotting

Lysates of GC-treated leukemic cells were prepared using 2× Laemmli buffer as mentioned earlier (Uddin et al., 2004). For protein quantification ND-1000 (NanoDrop Technologies, Thermo Scientific, United States) was used. After adding reducing agent cell lysates were resolved using SDS–PAGE and transferred to polyvinylidene difluoride (PVDF) membrane (Immobilon, Millipore, Billerica, MA, United States). Membranes were then probed with various antibodies as described previously (Uddin et al., 2004). Development and visualization of blots were done using ChemiDoc System (Amersham, Bio-Rad, United States).

### Measurement of Mitochondrial Membrane Potential

K562 and U937 cells were treated with different concentrations of GC for 24 h. Cells were stained with JC-1 and mitochondrial membrane potential was measured by flow cytometry using a BD LSRFortessa analyzer (BD Biosciences, NJ, United States) (Iskandarani et al., 2016).

### Assay for Release of Cytochrome c

K562 and U937 cells were treated with and without GC and resuspended in hypotonic buffer after harvesting by centrifugation. Cytosolic and mitochondrial protein fractions were isolated by using protocol as described earlier (Uddin et al., 2005). Cytosolic fraction of K562 and U937 was resolved using 12% SDS–PAGE and immunoblotted with anti-cytochrome c and GAPDH (Uddin et al., 2005).

### Measurement of Mitochondrial Superoxide

K562 and U937 cell lines were treated with increasing concentrations of GC for 24 h. Cells were harvested and washed with HBSS. Cells were stained with 5 μM MitoSOX Red Mitochondrial Superoxide Indicator (Invitrogen, MA, United States) in HBSS for 20 min at 37°C and analyzed by flow cytometry (Ex: 488, Em: 575/26) to quantify the levels of mitochondrial superoxide (Syn et al., 2017).

### Measurement of Reactive Oxygen Species

K562 and U937 were treated with increasing doses of GC for 24 h, and washed with HBSS after harvesting. The cells were stained with 5 μM CellROX^TM^ Green Reagent (Invitrogen, MA, United States) in HBSS for 30 min at 37°C, washed twice and analyzed by flow cytometry (Ex: 488, Em: 530/30) to quantify the levels of ROS (Weidner et al., 2016).

### Measurement of Reduced Glutathione

Cells were treated with GC, harvested, and washed with HBSS. The cells were then stained with 10 μM ThiolTracker^TM^ Violet (Invitrogen, MA, United States) in HBSS for 30 min at 37°C. Cells were washed twice and analyzed by flow cytometry (Ex: 405, Em: 525/50) to quantify the levels of reduced glutathione (GSH) (Morzadec et al., 2014).

### Statistical Analysis

Data are expressed as the mean ± standard deviation (*SD*). Paired student’s *t*-test was used for statistical comparisons between various experimental groups. GraphPad Prism (version 7.0 for Windows, GraphPad Software Inc., San Diego, CA, United States^1^) was used for all statistical analysis and for figure generation. Values of ^∗^*P* ≤ 0.05 and ^∗∗^*P* ≤ 0.001 reflected to be statistically significant.

## Results

### Isolation and Characterization of GC From Aquatic Fungus

Greensporone C (GC) was isolated as a colorless compound from organic fraction as mentioned above. Using HRESIMS, molecular formula for GC was assigned as C_19_H_25_O with molecular weight of 319.15. Purity of isolated compound was established using UPLC and was found to be >98% (El-Elimat et al., 2014).

### GC Inhibits Cell Proliferation and Induces Apoptosis in Leukemia Cell Lines

**FIGURE 2:**
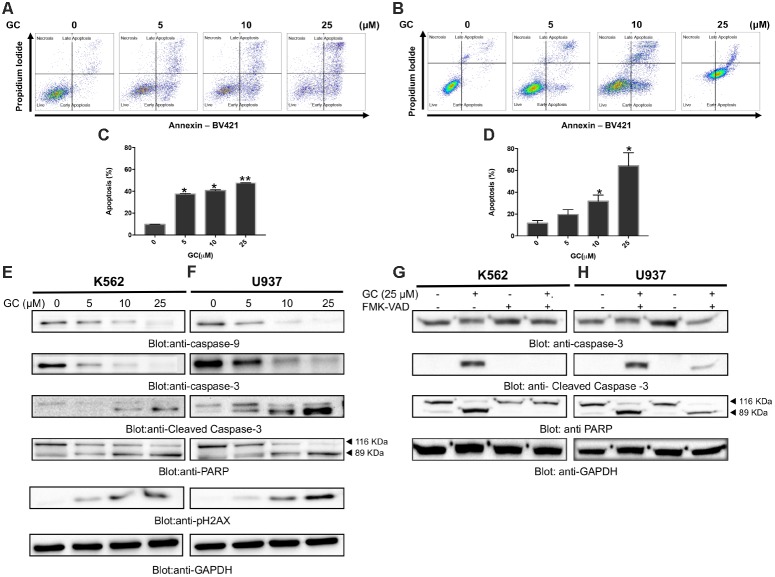
Greensporone C induces apoptosis in leukemic cells. K562 **(A)** and U937 **(B)** cells were treated with GC as indicated and analyzed by flow cytometry. GC significantly induced apoptosis in dose-dependent manner in K562 **(C)** and U937 **(D)**. The graph displays the mean ± SD of three independent of experiments. ^∗^*P* < 0.05 and ^∗∗^*P* < 0.001. GC-mediated activation of the caspase cascades in K562 **(E)** and U937 **(F)** cells. z-VAD-FMK reversed GC-induced caspase activation in K562 **(G)** and U937 **(H)** cells. Cells were treated with GC and z-VAD-FMK alone and in combination for 24 h. At the end of 24 h they were lysed and immunoblotted with antibodies against caspase-9, caspase-3, cleaved caspase-3, PARP, and GAPDH.

Initially we sought to determine the effect of GC on panel of leukemic cell lines (K562, U937, and AR230). Cells were treated with increasing concentrations of GC for 24 h and MTT assay was performed to assess the viability. As shown in **Figure 1B**, 5, 10, 25, and 50 μM of GC resulted in significant reduction of cell viability in all the cell lines. At a dose of 50 μM, 10.0, 4.0, and 17.0% of cells were found to be viable in K562, U937, and AR230 cells, respectively, in comparison to control. GC did not show any effects on normal peripheral mononuclear cells (PBMC) (Supplementary Figure 1A). In subsequent experiments, cell cycle phase distribution analysis was performed using flow cytometry. As depicted in **Figures 1C–F**, the increase in subG0 population in GS-treated cells was accompanied with a decrease percentage of the cells in G0/G1, S, and G2/M phases compared to control, a feature of cells that undergo apoptosis (Healy et al., 1998; Huang et al., 2015). Annexin V/PI dual staining was further employed to confirm that GC-induced subG0 apoptotic fraction is indeed an apoptotic feature (**Figures 2A–D**). We next investigated the functional role of GC on caspase activation in leukemic cells. Western blot analysis revealed that GC treatment of leukemic cell activates caspase-9, and caspase-3 in a dose-dependent manner. Consistent with this cleavage of PARP was also increased with the increase in GC concentrations. An increase in γ-H2AX-a marker for DNA double-stranded breakage was observed in response to GC treatment in leukemic cells suggesting a role of DNA damage in GC-mediated apoptosis (**Figures 2E,F**). Moreover, GC-mediated caspase-3 activation and PARP cleavage were reversed in leukemic cells pretreated with 20 μmol/l z-VAD-FMK, a pan caspase inhibitor (**Figures 2G,H**), demonstrating that the apoptosis triggered by GC was caspase dependent (Prabhu et al., 2017). In addition we also performed a combination study with imatinib, a well-known anticancer agent to determine whether GC can potentiate the apoptotic effects of imatinib in leukemic cells. K562 cells were treated with subtoxic doses of GC (10 μM) and imatinib (1 μM) for 24 h. As shown in Supplementary Figure, GC treatment of K562 potentiated imatinib-induced apoptotic effects (Supplementary Figures 1B,C).

### GC Treatment of Leukemia Cells Causes Suppression of Constitutive AKT and Its Associated Signaling Pathways

**FIGURE 3:**
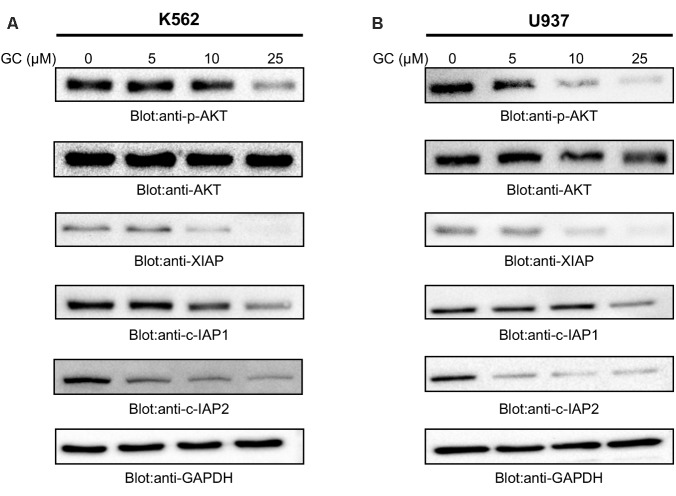
Greensporone C dephosphoryalted AKT and suppresses the expression of downstream targets. K562 **(A)** and U937 **(B)** cells were treated with increasing doses of GC for 24 h as indicated. After cell lysis, equal amounts of proteins were separated by SDS–PAGE, transfered to PVDF membrane, and immunoblotted with antibodies of p-AKT, AKT, XIAP, c-IAP1, c-IAP2, and GAPDH as indicated.

Previous reports have shown link between deregulated PI3-kinase/AKT signaling pathways and sustained survival of cells in hematological malignancy (Hussain et al., 2011, 2017). Furthermore, it has been shown that aberrant activation of AKT leads to oncogenic affects via activation of protein kinases and overexpression of antiapoptotic proteins (Rivera Rivera et al., 2016). We therefore, next determined the effect of GC on constitutively activated AKT. As shown in **Figures 3A,B** that GC treatment of leukemic cells resulted in dephosphorylation of AKT at Ser473 in a dose-dependent manner without affecting AKT protein levels. These findings support the notion that GC-mediated inhibition of cell viability is mediated through inactivation of AKT signaling pathway.

In the past few years IAP family members have been gaining importance in cancer treatment due to their ability to overexpress themselves in comparison to other deregulated proteins. High levels of IAPs promote survival of cancerous cells by suppressing apoptotic process (Hassan et al., 2014; Rathore et al., 2017). Accumulated evidences have indicated existence of correlation between AKT and XIAP. Phosphorylation of XIAP by AKT inhibits autoubiquitination and degradation process of XIAP thereby preventing apoptosis induced by caspase activation and conferring resistance to chemotherapeutic agents (Dan et al., 2004). Moreover, IAP family members have been found to be upregulated in cells when a constitutively active AKT is transfected into the cell (Jo et al., 2017) suggesting the link between AKT and IAP proteins. Therefore, we sought to determine whether GC-mediated suppression of AKT activity affects the expression of IAPs family members. Our results confirmed the ability of GC in suppressing expression of XIAP, cIAP-1, and cIAP-2 in both the cell lines. Altogether, western blot analysis conclude about involvement of IAP proteins in GC-mediated apoptosis.

### GC Treatment Suppresses Bcl-2 Expression and Enhances Bax/Bcl-2 Ratio in Leukemic Cells

**FIGURE 4:**
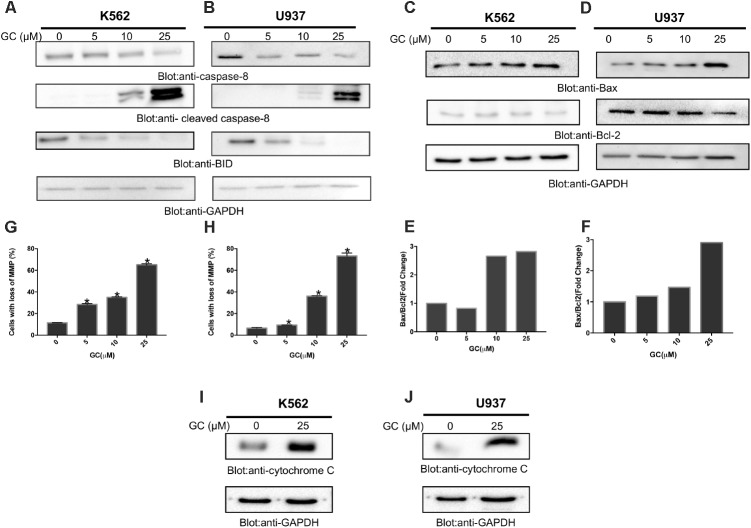
Greensporone C-induced mitochondrial signaling pathways in leukemic cells. GC treatment causes alteration in Bcl-2 expression. K562 **(A)** and U937 **(B)** cells were treated with increasing doses of GC for 24 h as indicated. After cell lysis, equal amounts of proteins were separated by SDS–PAGE, transfered to PVDF membrane, and immunoblotted with antibodies against caspase-8, cleaved caspase-8, Bid, and GAPDH as indicated. K562 **(C)** and U937 **(D)** cells were treated with GC as indicated and expression level of Bax and Bcl-2 was determined by immunoblotting with antibodies against Bax, Bcl-2, and GAPDH. Data obtained from immunoblot analyses of Bax and Bcl-2 in leukemic cell lines were used to evaluate the effects of GC on Bax/Bcl-2 ratio. Densitometric analysis of Bax and Bcl-2 bands in K562 **(E)** and U937 **(F)** cells was performed using AlphaImager Software (San Leandro, CA, United States), and data (relative density normalized to GAPDH) were plotted as Bax/Bcl-2 ratio. GC treatment causes the loss of mitochondrial membrane potential in leukemic cells. K562 **(G)** and U937 **(H)** cells were treated with indicated doses of GC for 24 h. After JC1 staining cells were analyzed by flow cytometry as described in the section “Materials and Methods.” The graph displays the mean ± SD of three independent of experiments. ^∗^*P* < 0.05 and ^∗∗^*P* < 0.001. GC-induced release of cytochrome c. K562 **(I)** and U937 **(J)** cells were treated with and without GC for 24 h. Cytoplasmic fraction was isolated as described in the section “Materials and Methods.” Cell extracts were separated on SDS–PAGE, transferred to PVDF membrane, and immunoblotted with an antibody against cytochrome c and GAPDH.

The apoptotic signaling is initiated by activation of caspase-8 that leads to activation of Bid and its translocation to mitochondrial membrane for its pro-apoptotic functions (Uddin et al., 2008). As shown in **Figures 4A,B**, treatment of K562 and U937 cells with GC resulted in activation of caspase 8 with subsequent decreased level of bid, a notion for its truncation. The truncated Bid is involved in regulation of Bax, a member Bcl-2 family (Anto et al., 2002). Bcl-2 family members play a significant and pivotal role in regulating apoptosis by maintaining a balance between anti-apoptotic molecules such as Bcl-2 and pro-apoptotic molecule Bax. Slight imbalance or disturbance in their levels leads to induction or inhibition of cell death (Martinou and Youle, 2011). Therefore, the effect of GC treatment on the expression levels of Bax and Bcl-2 in the leukemic cell lines was evaluated. As shown in **Figures 4C,D** treatment of leukemic cells with GC caused a decrease in expression levels of anti-apoptotic Bcl2 protein with subsequent increase in expression level of pro-apoptotic protein Bax. Raisova et al. (2001) reported that low Bax/Bcl-2 ratio is associated with resistant cells whereas high Bax/Bcl-2 ratio is considered as characteristic feature for sensitive cells that may undergo apoptosis on drug treatment. In our study, the densitometric analysis revealed an increase in the Bax/Bcl-2 ratio in both cell lines (**Figures 4E,F**). These results are in concordance with findings of Raisova et al. (2001) and others (Thomas et al., 1996; Pepper et al., 1997; Raisova et al., 2001).

### GC-Mediated Activation of Mitochondrial Apoptotic Pathways in Leukemic Cells

The effect of GC on mitochondrial membrane potential was examined using JC1 stain. K562 and U937 cells were treated with 10, 25, and 50 μM GC for 24 h. Treatment of leukemic cells with GC resulted in loss of mitochondrial membrane potential as measured by JC1-stained green fluorescence, depicting apoptotic cells (**Figures 4G,H** additional images representing loss in mitochondrial membrane potential are shown in Supplementary Figures 2A,B). It has been shown that loss of mitochondrial membrane potential can induce the release of cytochrome c from mitochondria to the cytosol during the apoptosis (Cai et al., 2001; Looi et al., 2013). We sought to determine whether GC treatment of leukemic cells could cause the release of cytochrome c from mitochondria to cytosol in K562 and U937. As shown in **Figures 4I,J**, treatment of K562 and U937 cells by GC-resulted cytochrome c expression was markedly increased as visualized and analyzed by western blot analysis. An increase in the band intensity of cytochrome c in cytosolic fraction suggests that GC treatment of leukemic cells causes apoptosis via intrinsic apoptotic pathway (Chandra et al., 2002).

### GC-Mediated Generation of ROS in Leukemic Cells

**FIGURE 5:**
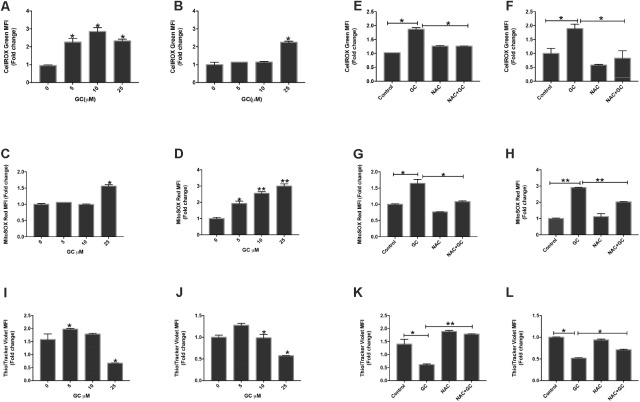
GC increases ROS generation in leukemic cells. K562 **(A,C)** and U937 **(B,D)** were treated with GC for 24 h. Cellrox and mitoSOX assays were performed to determine the level of ROS by flow cytometry as described in the section “Materials and Methods.” The graph displays the mean ± SD (standard deviation) fold change release of ROS of three experiments. *P* < 0.05, ^∗∗^*P* < 0.001. Effect of NAC on GC-induced generation of ROS. K562 **(E,G)** and U937 **(F,H)** were pre-treated with 10 mM NAC, subsequently treated with 25 μM GC for 24 h. Cellrox and mitoSOX assays were performed as described in the section “Materials and Methods.” The graph displays the mean ± SD fold change release of ROS of three experiments. *P* < 0.05, ^∗∗^*P* < 0.001. Effect of GC on GSH levels in leukemic cell lines. K562 **(I)** and U937**(J)** cells were treated with GC for 24 h and GSH level was determined by using ThioTracker assay kit and level of GSH level determined by flow cytometry. NAC pre-treated pre-ALL cell prevented GC-induced depletion of GSH in K562 **(K)** and U937 **(L)** cells. K562 and U937 cells were pretreated with 10 mM NAC, subsequently treated with 25 μM GC as indicated for 24 h and GSH content was determined by ThioTracker assay kit and level of GSH level determined by flow cytometry. The graph represents mean ± SD, ^∗^*P* < 0.05, ^∗∗^*P* < 0.001.

Chemotherapeutic compounds play an important role through generation of ROS which subsequently causes apoptosis in various cancer cells (Bhattacharyya et al., 2014; Gundala et al., 2014; Panda et al., 2014). ROS are highly reactive molecules which play important role in the maintenance and regulation of normal cell proliferation and differentiation. However, increase in levels of ROS is directly related to increased DNA damage and increased protein levels that causes cells to undergo apoptosis (Zhou et al., 2014). Besides this, attack on membrane phospholipids along with a loss in mitochondrial membrane potential occurs when an excess amount of ROS are produced in mitochondria, which ultimately releases apoptosis-inducing factors followed by caspase cascade activation and nuclear condensation (Lee et al., 2014; Liang et al., 2016). Generation of ROS is regarded as a prime factor for mitochondrial-dependent apoptosis. In line of these findings we investigated the role of GC in inducing ROS at the cellular level using Cell ROX assay by flow cytometry. K562 and U937 cells were treated with increasing doses of GC for 24 h. GC-treated leukemic cells resulted increase levels of ROS at the cellular level. The rise in intracellular levels of ROS is directly proportional to the amount of damage caused to the cellular components including mitochondria (Trachootham et al., 2009; Hamanaka and Chandel, 2010; Wellen and Thompson, 2010). Thus, we next thought to explore ROS-induced mitochondrial dysfunction using an oxidant-sensitive fluorescent dye, Mito-SOX red. Increase in the intensity levels of red fluorescence in MitoSOX assay corresponds to increasing $O_{2}^{-}$ levels in leukemic-treated cells (Maharjan et al., 2014). **Figures 5A–D** depict increased levels of ROS in leukemic cells as confirmed by cellROX and mitoSOX assay (Lee et al., 2017). Overall, the data showed that the role of GS in inducing ROS generation at cellular and mitochondrial levels was accompanied by apoptosis. NAC possesses the ability to inhibit oxidative stress by scavenging ROS directly. In the next experiment K562 and U937 cells were pretreated with NAC for 1 h followed by 25 μM GC. NAC abolished ROS generated by GC in both cell lines (**Figures 5E–H**). The cellRox and MitoSox data were in accord with each other.

### Effect of GC on Glutathione Level in Leukemic Cell Lines

Normal cells have the competence to detoxify themselves from various damaging oxidizing agents by possessing antioxidant enzyme and nonenzymatic antioxidants. One such antioxidant is GSH, which possesses multi-facet functions like cell differentiation, proliferation, and apoptosis. Imbalance in GSH levels is reported in many diseases including cancer. Substantial evidences available indicates increased GSH levels confer to chemo-resistance in tumor cells whereas decreased levels can sensitize cells toward cell death (Traverso et al., 2013). We, therefore, evaluated whether GC treatment of K562 and U937 cells reduced GSH level in leukemic cells. GC-treated leukemic cells exhibited significant depletion of GSH in a dose-dependent manner. NAC is one of the most commonly used synthetic precursors of cysteine and GSH and scavenges free radical via increasing GSH levels thereby attributing to its anti-ROS activity (Halasi et al., 2013). Pretreatment of leukemic cells with NAC followed by GC treatment significantly reversed GC-induced depletion of GSH level in K562 and U937 cells (**Figures 5I–L**).

### GC-Mediated ROS Generation Involved in Apoptotic Cell Death in Leukemic Cells

**FIGURE 6:**
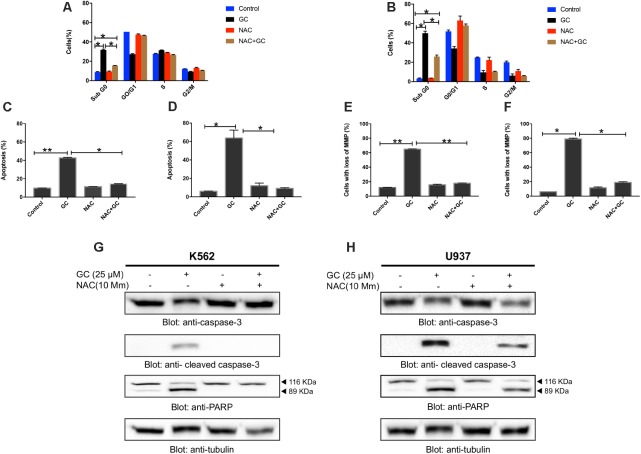
GC-mediated generation of ROS causes apoptosis in leukemic cells. NAC pre-treated leukemic cells prevented GC-induced increase in sub-G0 fraction in K562 **(A)** and U937 **(B)** cells. K562 and U937 cells were pretreated with 10 mM NAC, subsequently treated with 25 μM GC as indicated for 24 h, and cell cycle fraction was measured by flow cytometry. The graph displays the mean ± SD of three independent of experiments. ^∗^*P* < 0.05 and ^∗∗^*P* < 0.001. NAC pre-treated leukemic cell prevented GC-induced apoptosis. K562 **(C)** and U937 **(D)** cells were pretreated with 10 mM NAC, subsequently treated with 25 μM GC as indicated for 24 h and apoptosis was measured by staining with fluorescein-conjugated annexin-V and propidium iodide (PI) and analyzed by flow cytometry. The graph displays the mean ± SD of three independent of experiments. ^∗^*P* < 0.05 and ^∗∗^*P* < 0.001. NAC pre-treated leukemic cells prevented GC-mediated loss of mitochondrial membrane potential. K562 **(E)** and U937 **(F)** cells were pretreated with 10 mM NAC, subsequently treated with 25 μM GC as indicated for 24 h and loss of mitochondrial membrane potential was measured by JC1 staining and flow cytometry. The graph displays the mean ± SD of three independent of experiments. ^∗^*P* < 0.05 and ^∗∗^*P* < 0.001. NAC pre-treated leukemic cells prevented GC-mediated activation of caspases. K562 **(G)** and U937 **(H)** cells were pretreated with 10 mM NAC, subsequently treated with 25 μM GC as indicated for 24 h and lysed cell extracts were separated on SDS–PAGE, transferred to PVDF membrane, and immunoblotted with an antibody against procaspase-3, cleaved caspase-3, PARP, and tubulin.

Our experimental data provided insight into GC-induced apoptosis through ROS generation, we sought to determine whether ROS is involved in GC-induced leukemic cell death. As shown in **Figures 6A–F** (additional images are shown as Supplementary Figures 3A–F), treatment of K562 and U937 cell with GC significantly induced level SubG0 fraction of cell cycle, annexin V/PI staining, and loss of mitochondrial membrane potential in leukemic cell lines whereas pretreatment with 10 mM NAC markedly abrogated the GC-induced effects. Furthermore, NAC pretreatment of leukemic cells also prevented GC-induced activation of caspases and PARP cleavage (**Figures 6G,H**). This observation strongly implicates that GC-induced apoptosis in leukemic cells is mediated by generation of ROS.

## Discussion

Natural products have been gaining recognition and are becoming a significant part of research in the area of drug development and discovery. Natural products continue to offer a diverse source of bioactive compounds for drug discovery (Kingston, 2011; Newman and Cragg, 2016). Greensporone C (GC) is a resorcylic acid lactones that was isolated from a freshwater aquatic fungus *Halenospora* sp. (El-Elimat et al., 2014). We have previously shown that GC suppressed growth of MDA-MB-435 and HT-29 cells (El-Elimat et al., 2014). However, the antiproliferative effects GC and its underlying mechanism on leukemic cells have not been reported. In this study our results showed that GC induced a dose-dependent cytotoxic effects in leukemic cell lines. Our findings indicated that the inhibitory effect of GC on the growth of leukemic cells might be contributed by the induction of cell cycle arrest. Cell cycle analysis data revealed that treatment of leukemic cells with GC resulted in a significant increase in apoptotic (sub G0) phase. These are the features of a cell that is undergoing apoptotic cell death (Hussain et al., 2006; Vignon et al., 2013). In subsequent our data showed an increased Annexin/PI staining supporting that GC-mediated cytotoxic effects are due to induction of apoptosis. Caspases are hallmark of apoptosis that plays a critical role in execution of apoptosis (MacKenzie and Clark, 2008; Hensley et al., 2013).

Among caspase family caspase-3 has been shown to be a key component of the apoptotic machinery (Nunez et al., 1998). Caspase-3 is activated in apoptotic cells and cleaves several cellular proteins, including PARP. The cleavage of PARP is a hallmark of apoptosis by various antitumor agents (Duriez and Shah, 1997). In our study, we show that GC treatment of leukemic cells activates caspase-3 and cleaves PARP in a dose-dependent manner. In addition, our data also found that GC treatment phosphorylated H2AX, a marker for double-stranded break which can lead DNA degradation and eventually apoptotic cell death (Oben et al., 2017). Blocking of caspase activity by pretreatment with a pan caspase inhibitor, zVAD-fmk prevented GC-induced caspase-3 and PARP. These finding are strongly suggesting that GC-mediated apoptotic cell death in leukemic cells and the GC-induced apoptosis is caspase dependent.

The PI3k/AKT pathway plays a critical role in pro-survival signaling that prevents apoptotic cell death in various cancer cells (Uddin et al., 2006; Martelli et al., 2011; Brown and Banerji, 2017). High level of constitutive AKT activity has been linked with shorter survival of patients in many cancers including hematological malignancies (Uddin et al., 2006; Silva et al., 2008). Our data showed that leukemic cells possess constitutive/basal AKT activity (phosphorylated AKT) and treatment of with these cells with GC suppresses phosphorylated AKT without affecting the AKT proteins suggesting the role of AKT in survival and proliferation of leukemic cells. Activated AKT has been found to be involved in regulation of IAPs such as XIAP, c-IAP1, and c-IAP2 (Gagnon et al., 2003; Seol, 2008; Jo et al., 2017; Prabhu et al., 2018). Interestingly, XIAP has been shown to be a physiologic substrate of AKT, that inhibit programmed cell death and is directly involved in inhibition of caspase-3 and caspase activity (Dan et al., 2004; Rada et al., 2018). In this study we found that GC treatment of K562 and U937 leukemic cells down regulated the expression of XIAP, c-IAP1, and c-IAP2 in a dose-dependent manner. These findings suggest that GC-mediated apoptosis occurs via inactivation of AKT activity and downregulation of cIAPs in leukemic cells.

Apoptosis or program cell death is an important event that eliminates harmful and undesired cells to maintain the balance between survival and cell death (Vasilikos et al., 2017). There are two apoptotic pathways, the extrinsic or receptor-mediated apoptotic that involved death receptors and the intrinsic where mitochondrial signaling plays a major role. Most of the anticancer agents induced apoptosis via mitochondrial or intrinsic apoptotic pathway (Elmore, 2007; Lopez and Tait, 2015). Bcl-2 family members consist of pro-apoptotic molecule like Bax, Bak, and anti-apoptotic members like Bcl-2, Bcl-xL, etc. These members are known to play a significant and crucial role in mitochondrial-mediated apoptotic pathway or commonly known as intrinsic pathway (Wei et al., 2001; Opferman and Kothari, 2018). In the present investigation, we observed that GC treatment upregulated Bax expression and diminished Bcl-2 expression in K562 and U937 cells. Elevated Bax/Bcl2 ratio by GC resulted in a loss of mitochondrial potential and release of cytochrome c from mitochondria to cytosol in leukemic cells. It has been reported that complex apoptosome are formed by cytochrome C, apoptosome protease activating factor (APAF-1) and caspase-9 leading to cleavage of caspase-9 and its activation. Then activated caspase activates caspase-3 and intracellular PARP cleavage resulting in execution apoptotic cell death (Jin and El-Deiry, 2005). Our study is in concordance with these findings showing GC-induced activation of caspase-9, caspase-3, and cleavage of PARP suggesting that GC induced its apoptotic effects via intrinsic pathway.

It is generally believed that increasing amounts of ROS and resulting oxidative stress play an important role as a regulator of apoptosis in various cancer cell types (Dayem et al., 2010). Excessive ROS can cause cellular damage and lead to activation of multiple death pathways, such as mitochondrial, death receptor, and ER pathways of apoptosis (Redza-Dutordoir and Averill-Bates, 2016). Many natural and synthetic agent/s have been developed that induced apoptosis through ROS-mediated cell damage. Consistent with this, our data generated by using various experimental procedures showed a dose-dependent increase in ROS with subsequent decline in intracellular GSH content and mitochondrial membrane potential. Depletion in levels of GSH increased susceptibility to ROS resulting in DNA fragmentation (Yeh et al., 2012). Furthermore, we also showed that GC-induced proapoptotic effects is ROS dependent as pre-exposure of leukemic cells with NAC, a ROS scavenger prevented GC-induced loss of mitochondrial potential, GC-mediated caspase activation, and GC-induced PARP activation.

## Conclusion

Our finding demonstrates that GC-induced apoptosis occurs via inactivation of AKT and through suppressive effects on anti-apoptotic genes expression including XIAP, cIAPs, and Bcl-xL. Furthermore, GC treatment downregulated Bcl-2 expression with an increase in expression level of Bax resulting in collapse of mitochondrial integrity with release of cytochrome c. Released cytochrome c caused activation of pro-caspase-9, -3, and cleavage of PARP resulting in activation of intrinsic apoptotic pathways in leukemic cells. GC-induced its anticancer potential via ROS production. Leukemic cells pre-treated with NAC prevented GC-induced depletion of GSH as well as mitochondrial-caspase-induced apoptosis. Taken together, our results show that GC modulates the apoptotic response of human leukemic cells and raises the possibility of its use as a novel therapeutic strategy for hematological malignancies.

## Author Contributions

SU and KP designing of experiments, analysis of data, and manuscript writing. KP, KS, AK, and SK designing of experiments, data analysis, manuscript writing, and editing. MM, AI, SD, and HO provided support in maintenance of cell culture and proof read of the manuscript. TE-E, NO, and FA isolated GC from fungal strain.

## Conflict of Interest Statement

The authors declare that the research was conducted in the absence of any commercial or financial relationships that could be construed as a potential conflict of interest.

## Footnotes

1 http://www.graphpad.com
