# Supplementary material for: Greensporone C, a Freshwater Fungal Secondary Metabolite Induces Mitochondrial-Mediated Apoptotic Cell Death in Leukemic Cell Lines
Source: Front Pharmacol. 2018 Jul 16;9:720. doi: 10.3389/fphar.2018.00720 (PMC6054921; doi:10.3389/fphar.2018.00720)
Supplement: Supplementary file 1 [file Data_Sheet_1.pdf]

Supplementary Figure Legends

Supplementary Figure 1. Effect of GC on normal peripheral blood mononuclear cells (PBMC). PBMCs obtained from a healthy donor were treated with 10 and 25µM GC and MTT assays was performed as described in Methods (**A**). GC potentiate apoptotic effect of Imatinib in leukemic cells. K562 cells were treated with GC (10µM) and Imatinib (1µM) alone and in combination and analysed by flow cytometry **(B and C).** The graph displays the mean +/-SD of three independent of experiments *P < 0.05 and **P < 0.001. NS – Not significant.

Supplementary Figure 2. . GC treatment causes the loss of MMP in leukemic cells. K562 (**A**) and U937 (**B**) cells were treated with indicated doses of GC for 24 hours. After JC1 staining cells were analyzed by flow cytometry as described in Materials and Methods. The graph displays the mean +/-SD of three independent of experiments. *P <0.05 and **P < 0.001.

Supplementary Figure 3. NAC pre-treated leukemic cells prevented GC-induced increase in Sub G0 fraction in K562 (**A**) and U937(**B**) cells. K562 and U937 cells were pretreated with 10 mM NAC, subsequently treated with 25µM GC as indicated for 24 h and cell cycle fraction was measured by flow cytometry. The graph displays the mean +/-SD of three independent of experiments. *P <0.05 and **P < 0.001. NAC pre-treated leukemic cell prevented GC induced apoptosis. K562 (**C**) and U937(**D**) cells were pretreated with 10 mM NAC, subsequently treated with 25µM GC as indicated for 24 h and apoptosis was measured by staining with fluorescein-conjugated annexin-V and propidium iodide (PI) and analyzed by flow cytometry. The graph displays the mean +/-SD of three independent of experiments. *P <0.05 and **P < 0.001. NAC pre-treated leukemic cells prevented GC-mediated loss of MMP. K562 (**E**) and U937(**F**) cells were pretreated with 10 mM NAC, subsequently treated with 25µM GC as indicated for 24 h and loss of MMP was measured by JC1 staining and flow cytometry. The graph displays the mean +/-SD of three independent of experiments. *P <0.05 and **P < 0.001.
