# Supplementary figures and images for: Greensporone C, a Freshwater Fungal Secondary Metabolite Induces Mitochondrial-Mediated Apoptotic Cell Death in Leukemic Cell Lines
Source: Front Pharmacol. 2018 Jul 16;9:720. doi: 10.3389/fphar.2018.00720 (PMC6054921; doi:10.3389/fphar.2018.00720)

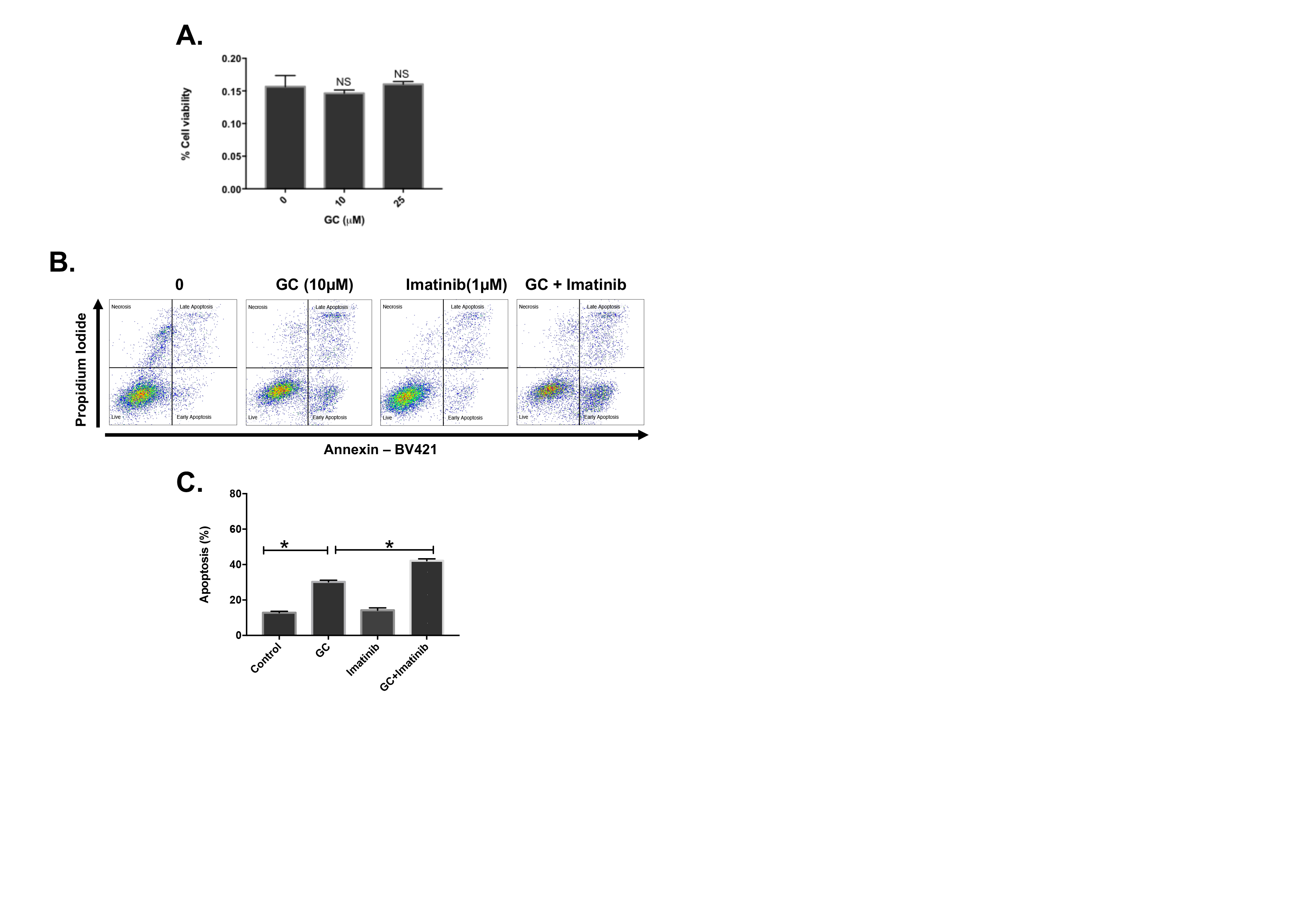

Supplement: Supplementary file 2 [file Image_1.tif]

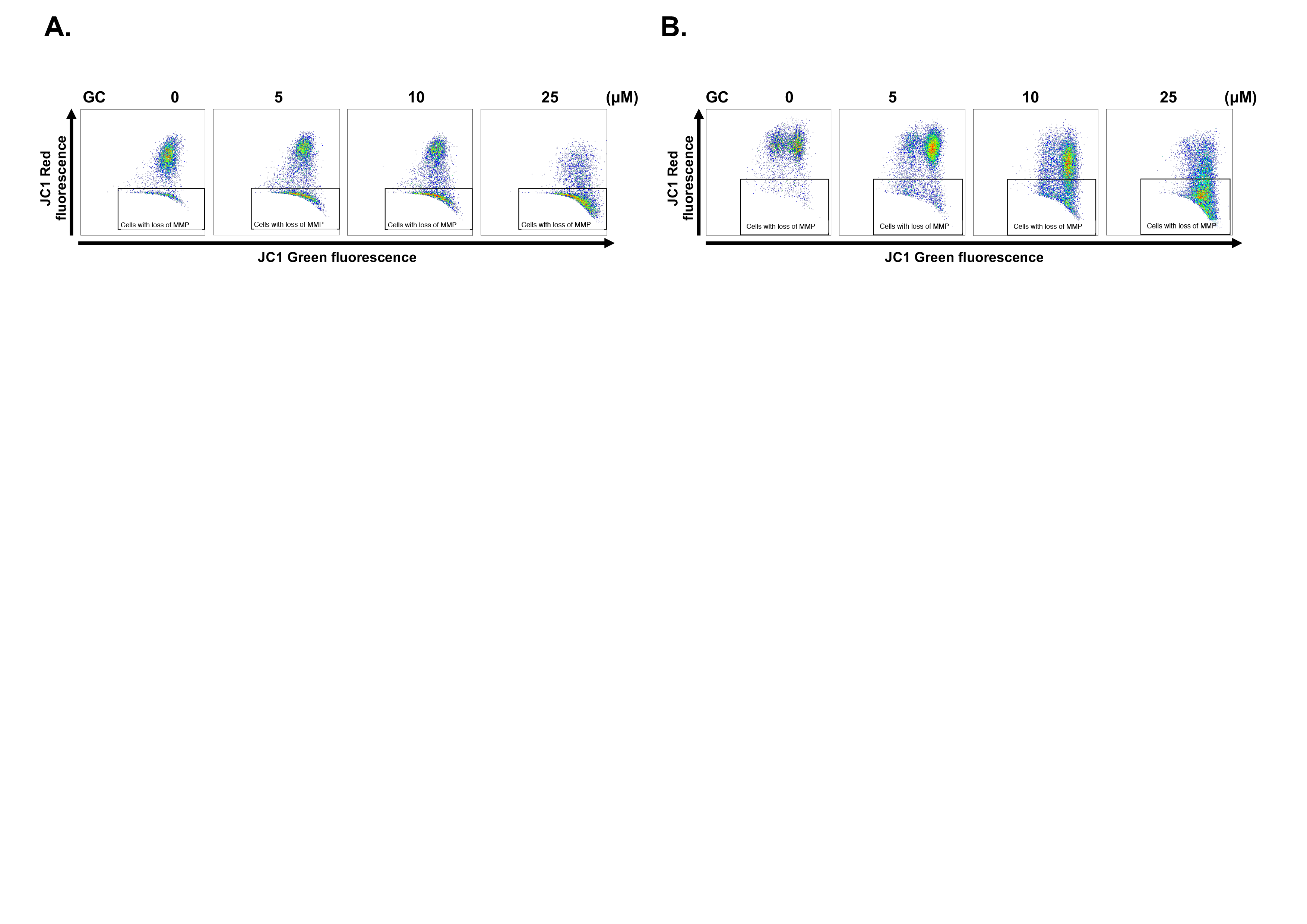

Supplement: Supplementary file 3 [file Image_2.tif]

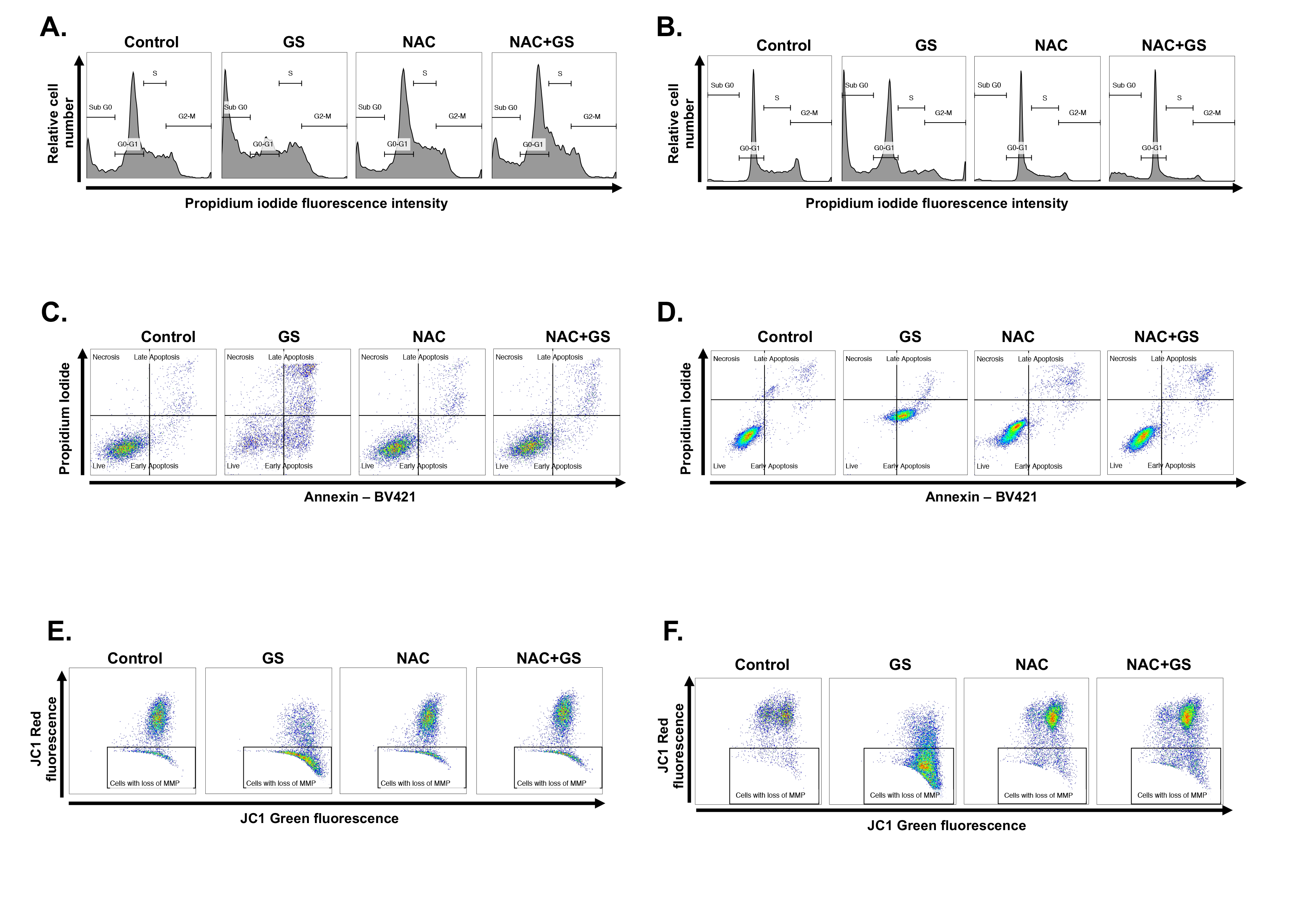

Supplement: Supplementary file 4 [file Image_3.tif]
